# Supplementary material for: Resolving the full spectrum of human genome variation using Linked-Reads
Source: Genome Res. 2019 Apr;29(4):635–45. doi: 10.1101/gr.234443.118 (PMC6442396; doi:10.1101/gr.234443.118)
Supplement: Supplemental Material [file supp_gr.234443.118_Supplemental_longranger-2.2.zip › longranger-2.2.tar.bz2!/lr2.2-public/lib/stan/cmdstan/stan/doc/api/html/agrad_2rev_2functions_2gamma__p_8hpp_source.html]

Stan: src/stan/agrad/rev/functions/gamma\_p.hpp Source File


|  |  |
| --- | --- |
|  | Stan  2.5.0  probability, sampling & optimization |


- Main Page
- Related Pages
- Namespaces
- Classes
- Files

- File List
- File Members

All Classes Namespaces Files Functions Variables Typedefs Enumerations Enumerator Friends Macros Pages

- src
- stan
- agrad
- rev
- functions

gamma\_p.hpp

Go to the documentation of this file.

1 #ifndef STAN\_\_AGRAD\_\_REV\_\_FUNCTIONS\_\_GAMMA\_P\_HPP

2 #define STAN\_\_AGRAD\_\_REV\_\_FUNCTIONS\_\_GAMMA\_P\_HPP

3

4 #include <valarray>

5 #include <stan/agrad/rev/var.hpp>

6 #include <stan/agrad/rev/internal/vv\_vari.hpp>

7 #include <stan/agrad/rev/internal/dv\_vari.hpp>

8 #include <stan/agrad/rev/internal/vd\_vari.hpp>

9 #include <stan/math/functions/gamma\_p.hpp>

10 #include <boost/math/special\_functions/gamma.hpp>

11 #include <boost/math/special\_functions/digamma.hpp>

12

13 namespace stan {

14  namespace agrad {

15

16  namespace {

17  class gamma\_p\_vv\_vari : public op\_vv\_vari {

18  public:

19  gamma\_p\_vv\_vari(vari\* avi, vari\* bvi) :

20  op\_vv\_vari(stan::math::gamma\_p(avi->val\_,bvi->val\_),

21  avi,bvi) {

22  }

23  void chain() {

24

25  double u = stan::math::gamma\_p(avi\_->val\_, bvi\_->val\_);

26

27  double S = 0.0;

28  double s = 1.0;

29  double l = std::log(bvi\_->val\_);

30  double g = boost::math::tgamma(avi\_->val\_);

31  double dig = boost::math::digamma(avi\_->val\_);

32

33  int k = 0;

34  double delta = s / (avi\_->val\_ \* avi\_->val\_);

35

36  while (std::fabs(delta) > 1e-6) {

37  S += delta;

38  ++k;

39  s \*= -bvi\_->val\_ / k;

40  delta = s / ((k + avi\_->val\_) \* (k + avi\_->val\_));

41  }

42

43

44  avi\_->adj\_ -= adj\_ \* ((u) \* ( dig - l ) + std::exp( avi\_->val\_ \* l ) \* S / g);

45  bvi\_->adj\_ += adj\_ \* (std::exp(-bvi\_->val\_) \* std::pow(bvi\_->val\_, avi\_->val\_ - 1.0) / g);

46  }

47  };

48

49  class gamma\_p\_vd\_vari : public op\_vd\_vari {

50  public:

51  gamma\_p\_vd\_vari(vari\* avi, double b) :

52  op\_vd\_vari(stan::math::gamma\_p(avi->val\_,b),

53  avi,b) {

54  }

55  void chain() {

56

57  double u = stan::math::gamma\_p(avi\_->val\_, bd\_);

58

59  double S = 0.0;

60  double s = 1.0;

61  double l = std::log(bd\_);

62  double g = boost::math::tgamma(avi\_->val\_);

63  double dig = boost::math::digamma(avi\_->val\_);

64

65  int k = 0;

66  double delta = s / (avi\_->val\_ \* avi\_->val\_);

67

68  while (std::fabs(delta) > 1e-6) {

69  S += delta;

70  ++k;

71  s \*= -bd\_ / k;

72  delta = s / ((k + avi\_->val\_) \* (k + avi\_->val\_));

73  }

74

75  avi\_->adj\_ -= adj\_ \* ((u) \* ( dig - l ) + std::exp( avi\_->val\_ \* l ) \* S / g);

76  }

77  };

78

79  class gamma\_p\_dv\_vari : public op\_dv\_vari {

80  public:

81  gamma\_p\_dv\_vari(double a, vari\* bvi) :

82  op\_dv\_vari(stan::math::gamma\_p(a,bvi->val\_),

83  a,bvi) {

84  }

85  void chain() {

86  bvi\_->adj\_ += adj\_ \* (std::exp(-bvi\_->val\_) \* std::pow(bvi\_->val\_, ad\_ - 1.0) / boost::math::tgamma(ad\_));

87  }

88  };

89  }

90

91  inline var gamma\_p(const stan::agrad::var& a,

92  const stan::agrad::var& b) {

93  return var(new gamma\_p\_vv\_vari(a.vi\_,b.vi\_));

94  }

95

96  inline var gamma\_p(const stan::agrad::var& a,

97  const double& b) {

98  return var(new gamma\_p\_vd\_vari(a.vi\_,b));

99  }

100

101  inline var gamma\_p(const double& a,

102  const stan::agrad::var& b) {

103  return var(new gamma\_p\_dv\_vari(a,b.vi\_));

104  }

105

106  }

107 }

108 #endif

stan::agrad::fabs

fvar< T > fabs(const fvar< T > &x)

**Definition:** fabs.hpp:18

dv\_vari.hpp

stan::agrad::tgamma

fvar< T > tgamma(const fvar< T > &x)

**Definition:** tgamma.hpp:15

stan::agrad::pow

fvar< T > pow(const fvar< T > &x1, const fvar< T > &x2)

**Definition:** pow.hpp:17

vd\_vari.hpp

stan::agrad::var::vi\_

vari \* vi\_

Pointer to the implementation of this variable.

**Definition:** var.hpp:40

var.hpp

stan::agrad::gamma\_p

fvar< T > gamma\_p(const fvar< T > &x1, const fvar< T > &x2)

**Definition:** gamma\_p.hpp:16

gamma\_p.hpp

stan::agrad::var

Independent (input) and dependent (output) variables for gradients.

**Definition:** var.hpp:27

stan::math::e

double e()

Return the base of the natural logarithm.

**Definition:** constants.hpp:86

stan::agrad::digamma

fvar< T > digamma(const fvar< T > &x)

**Definition:** digamma.hpp:16

vv\_vari.hpp

stan::agrad::log

fvar< T > log(const fvar< T > &x)

**Definition:** log.hpp:15

stan::agrad::exp

fvar< T > exp(const fvar< T > &x)

**Definition:** exp.hpp:16

stan::math::gamma\_p

double gamma\_p(double x, double a)

**Definition:** gamma\_p.hpp:53


---

[ Stan Home Page ]

*© 2011–2014,
Stan Development Team.*
